# *RNASEL* and *MIR146A* SNP-SNP Interaction as a Susceptibility Factor for Non-Melanoma Skin Cancer

**DOI:** 10.1371/journal.pone.0093602

**Published:** 2014-04-03

**Authors:** Shohreh F. Farzan, Margaret R. Karagas, Brock C. Christensen, Zhongze Li, Jacquelyn K. Kuriger, Heather H. Nelson, on behalf of the New Hampshire Skin Cancer Study

**Affiliations:** 1 Department of Community and Family Medicine, Section of Biostatistics and Epidemiology, Geisel School of Medicine at Dartmouth, Lebanon, New Hampshire, United States of America; 2 Norris Cotton Cancer Center, Geisel School of Medicine at Dartmouth, Lebanon, New Hampshire, United States of America; 3 Department of Pharmacology and Toxicology, Geisel School of Medicine at Dartmouth, Hanover, New Hampshire, United States of America; 4 Division of Epidemiology and Community Health, The Masonic Cancer Center, University of Minnesota, Minneapolis, Minnesota, United States of America; Sanjay Gandhi Medical Institute, India

## Abstract

Immunity and inflammatory pathways are important in the genesis of non-melanoma skin cancers (NMSC). Functional genetic variation in immune modulators has the potential to affect disease etiology. We investigated associations between common variants in two key regulators, *MIR146A* and *RNASEL*, and their relation to NMSCs. Using a large population-based case-control study of basal cell (BCC) and squamous cell carcinoma (SCC), we investigated the impact of *MIR146A* SNP rs2910164 on cancer risk, and interaction with a SNP in one of its putative targets (*RNASEL*, rs486907). To examine associations between genotype and BCC and SCC, occurrence odds ratios (OR) and 95% confidence intervals (95%CI) were calculated using unconditional logistic regression, accounting for multiple confounding factors. We did not observe an overall change in the odds ratios for SCC or BCC among individuals carrying either of the *RNASEL* or *MIR146A* variants compared with those who were wild type at these loci. However, there was a sex-specific association between BCC and *MIR146A* in women (OR_GC_ = 0.73, [95%CI = 0.52–1.03]; OR_CC_ = 0.29, [95% CI = 0.14–0.61], p-trend<0.001), and a reduction in risk, albeit not statistically significant, associated with *RNASEL* and SCC in men (OR_AG_ = 0.88, [95%CI = 0.65–1.19]; OR_AA_ = 0.68, [95%CI = 0.43–1.08], p-trend = 0.10). Most striking was the strong interaction between the two genes. Among individuals carrying variant alleles of both rs2910164 and rs486907, we observed inverse relationships with SCC (OR_SCC_ = 0.56, [95%CI = 0.38–0.81], p-interaction = 0.012) and BCC (OR_BCC_ = 0.57, [95%CI = 0.40–0.80], p-interaction = 0.005). Our results suggest that genetic variation in immune and inflammatory regulators may influence susceptibility to NMSC, and novel SNP-SNP interaction for a microRNA and its target. These data suggest that RNASEL, an enzyme involved in RNA turnover, is controlled by miR-146a and may be important in NMSC etiology.

## Introduction

Non-melanoma skin cancers (NMSC) are the most prevalent malignancy in the US, exceeding all other cancers combined with an estimated 2 million new diagnoses each year [1], [2]. Incidence of NMSC, which include basal cell (BCC) and squamous cell carcinomas (SCC), has continued to rise. Both BCC and SCC are relatively treatable and have low rates of mortality, but NMSCs can have high rates of recurrence and can cause significant disfiguration, particularly on the head and neck regions where they commonly occur [2]–[4]. While BCC and SCC both arise from keratinocytes or their precursors, there are key differences in their incidence and etiology. BCC tends to be more common and is thought arise *de novo*, while SCC develops in a multistep progression from premalignant precursor lesions to more aggressive skin tumors over time [2]–[4]. SCC also appears to be more strongly related to cumulative lifetime sun exposure and has a greater capacity to metastasize [5]. NMSCs are generally not included in cancer registries, making large epidemiological studies challenging.

While ultraviolet light exposure and skin sensitivity are known risk factors for NMSC development, inflammation and immunity are also key elements of NMSC etiology. Immunosuppressed individuals tend to have much higher NMSC incidence rates than immunocompetent individuals, as evidenced by 65- to 250-fold increased incidence rates of SCC and 10- to 16-fold increased incidence rates of BCC in organ transplant recipients who are routinely treated with immunosuppressive agents to prevent organ rejection, and a more modest increase in NMSC incidence among individuals chronically treated with glucocorticoids [6]–[9]. Further, imiquimod, a topical cream that is thought to induce a localized immune response, has been a successful treatment for NMSC and precancerous skin lesions [10]. Given that immune function has been closely linked to NMSC development, it is likely that genetic variation in key immune regulatory mechanisms impacts susceptibility to these prevalent malignancies.

MiR-146a is a microRNA (miRNA) of particular interest in the etiology NMSCs, as it is an important modulator of inflammatory immune responses, coordinating myeloid and lymphocyte function to impact aspects of both innate and adaptive immunity [11]. MiRNAs are short, non-coding RNAs that repress specific target mRNAs by binding imperfectly to sequences frequently located in 3′-untranslated regions (UTR), and have emerged as key regulators of virtually all cellular processes, both physiological and pathogenic. Post-transcriptional regulation by miRNAs is thought to affect the majority of mRNA transcripts and functional genetic variation in miRNAs has the potential to broadly impact disease processes, given the large number of genes and pathways targeted by each miRNA. However, there are only a few examples of functional polymorphisms in miRNAs. Among them is rs2910164 contained in the pre-miR-146a, which reduces miR-146a abundance, in turn altering the cellular transcriptome and increasing levels of its targets [12], [13]. rs2910164 may play a role in certain inflammatory conditions, such as rheumatoid arthritis and inflammatory bowel disease, and has been shown to increase susceptibility to hepatocellular carcinoma, and thyroid cancer [12], [14]–[16].

MiR-146a has 224 potential mRNA binding targets, including the cancer susceptibility gene *RNASEL* [17]. Ribonuclease L (RNASEL) is an interferon-activated ribonuclease, which degrades cellular and viral RNA upon activation. This activity is critical to cellular defense against viral infection, by limiting viral propagation and inducing apoptosis in infected cells, prior to a full immune response [18]. RNASEL is maintained at very low levels in the cell and its regulation is unclear, but may include miRNA suppression of its transcript and targeting of RNASEL by a miRNA, such as miR-146a, would serve to reduce cellular RNASEL levels [19], [20]. The *RNASEL* rs486907 Arg to Gln variant has 3-fold reduced enzyme activity, which could enhance virus susceptibility, diminish control of cellular RNA levels, impair the cellular stress response, or induce apoptosis [21]. While under normal conditions RNASEL has tumor suppressive and anti-proliferative functions, *RNASEL* variants, including the common rs486907 variant, have been associated with risk of a number of cancers, i.e. prostate, colorectal and pancreatic cancer, and overall risk of cancer in individuals of African descent [21]–[32].

To examine the effect of genetic variation in key immune components on NMSC susceptibility. we investigated the impact of the *MIR146A* SNP rs2910164 on NMSC risk, and potential interaction with one of its putative targets *RNASEL* (rs486907) as part of a large population-based, case-control study of BCC and SCC in New Hampshire.

## Materials and Methods

### Ethics Statement

All study protocols and materials were approved by the Dartmouth College Institutional Review Board (federal assurance number 00003095). All participants provided written, informed consent at the time of enrollment. All consent procedures and materials were approved by the Dartmouth College Institutional Review Board in accordance with the Committee for the Protection of Human Subjects at Dartmouth College.

### Study Population

Study subjects included those described in our earlier reports [33]–[36]. Briefly, to identify cases we enlisted the collaboration of dermatologists and pathology laboratories throughout New Hampshire and bordering regions [34]. Newly diagnosed cases of histologically confirmed BCC and SCC in New Hampshire were identified from July 1, 1993 to June 30, 1995 in the initial enrollment phase and July 1, 1997 to March 30, 2000 in the second enrollment phase. Eligible subjects included New Hampshire residents who, at the time of diagnosis, 1) were between 25 and 74 years of age, 2) had a listed telephone number and 3) spoke English. The BCC cases were randomly sampled in order to ensure representativeness of age, sex, and anatomic site for all incident BCCs within New Hampshire. We identified 1084 potential participants. Of these caess, we contacted and confirmed the eligibility on 1036 (96%), of whom 80% agreed to participate. Individuals with lesions on genital sites were excluded due to likely differences in etiology.

Controls aged 25–64 years were identified from the New Hampshire State Department of Transportation files and those aged 65–74 years were obtained from enrollment lists from the Center for Medicaid and Medicare Services. Potential controls were frequency-matched on age (25–34, 35–44, 45–54, 55–64, 65–69 and 70–74 years) and gender to the combined distribution of case groups (roughly a two to one ratio to cases in the first phase and one to one ratio in the second phase) [33], [34]. As with cases, controls were required to speak English and to have a listed telephone number. For interviewing purposes, controls were randomly assigned reference dates corresponding to the cases’ diagnosis dates. Of the 1527 potential controls, 1462 (96%) were contacted and confirmed as eligible, and 1066 (73%) of those were interviewed.

### Personal Interview

Study participants completed a structured personal interview, usually at their homes. To minimize reporting bias, we did not reveal the specific hypotheses of interest to either the interviewer or participant, and did not inform the interviewers of the case-control status of participants. The interview included sociodemographic information (level of education), tobacco use, prolonged use of glucocorticoid drugs (for one month or longer) and reasons for use, assessment of pigmentary characteristics and nevi, and questions relating to skin sensitivity to the sun and sun exposure using a standardized instrument developed for a case-control study conducted in Australia [37], [38].

### Genotyping

We collected a venous blood sample (20–30 ml) in heparinized tubes and separated plasma, white blood cells and red blood cells by centrifugation at 3000 rpm for 20 min at 4°C. Cells were washed twice in saline, aliquoted and stored at −80°C until analysis. Each specimen was labeled and given a unique identifier that did not reveal the subject’s case-control status. DNA was extracted using Qiagen Genomic DNA extraction kits (Valencia, CA). For quality assurance purposes, 10% of blood and buccal samples were used as integrated duplicates. Genotyping for the *MIR146A* SNP (rs2910164) was done using an allelic discrimination assay. The *RNASEL* SNP (rs486907) was genotyped at the University of Minnesota Biomedical Genomics Center using the Sequenom platform.

### Statistical Analysis

We classified cases according to their status as of the date of their first skin cancer diagnosed during the study period, or for controls, as of their reference date. This subject classification plan results in relative risk estimates of incidence density ratios [39]. We examined risk of both BCC and SCC according to *MIR146A* and *RNASEL* genotypes, in comparison to control subjects. We examined the main effects for each genotype, as well as the statistical interaction between the genotypes, computing the adjusted odds ratios (OR) and 95% confidence intervals (CI) of SCC and BCC associated with *MIR146A* and *RNASEL* genotypes. In each of these analyses, we used unconditional logistic regression, taking into account multiple confounding factors [40]. These covariates included age at diagnosis, sex (except in analyses stratified by sex), level of education, skin sensitivity to the sun, and lifetime number of painful sunburns. Analyses of SCC additionally included adjustment for cigarette smoking status 1 year before the reference date, as smoking been found to increase risk of SCC, but not BCC [41]. Statistical analyses were conducted with SAS 9.2 (SAS Institute, Cary, NC). All *P* values are two sided.

## Results

Our study included 920 BCC cases, 682 SCC cases, and 824 controls, with men comprising the majority of all groups (Table 1). The mean age of our study population was 61.4 years. More than two-thirds of participants were current or former smokers (n = 1524), with BCC cases more likely to be never smokers (p = 0.002) than controls. Approximately half of all participants (n = 1164) indicated having three or more severe sunburns in their lifetime, with greater number of lifetime sunburns reported among both BCC and SCC cases, as compared to controls (BCC: p<0.001, SCC: p<0.001). Greater skin sensitivity, as indicated by tendency to burn, was also reported more frequently for both BCC (p<0.001) and SCC (p<0.001), as compared to controls.

**Table 1:** Characteristics of Participants in New Hampshire Skin Study*.

		N (%) or mean		
Variable	Overall	Control	BCC	SCC
*Sex*				
Men	1459	504 (34.5)	518 (35.5)	437 (30.0)
Women	967	320 (33.1)	402 (41.6)	245 (25.3)
*Age (years)*				
Mean	61.4	61.6	59.1	64.2
25–34	26	5 (19.2)	18 (69.2)	3 (11.5)
35–49	345	115 (33.3)	181 (52.5)	49 (14.2)
50–54	205	56 (27.3)	102 (49.8)	47 (22.9)
55–59	289	103 (35.6)	111 (38.4)	75 (26.0)
60–64	356	115 (32.3)	142 (39.9)	99 (27.8)
65–69	587	238 (40.6)	171 (29.1)	178 (30.3)
70–74	618	192 (31.1)	195 (31.6)	231 (37.4)
*Education*				
High School, GED ortechnical school	1005	388 (38.6)	331 (33.0)	286 (28.5)
College	839	265 (31.6)	340 (40.5)	234 (27.9)
Postgraduate School	580	171 (29.5)	247 (42.6)	162 (27.9)
*Smoking*				
Never	901	285 (31.6)	393 (43.6)	223 (24.8)
Former	1169	409 (35.0)	405 (34.6)	355 (30.4)
Current	355	130 (36.6)	121 (34.1)	104 (29.3)
*Skin Sensitivity* †				
Severe sunburn withblistering	172	44 (25.6)	61 (35.5)	67 (39.0)
Painful sunburnfollowed by peeling	773	202 (26.1)	329 (42.6)	242 (31.3)
Mild sunburn withsome tanning	1181	422 (35.7)	455 (38.5)	304 (25.4)
Tan without sunburn	297	155 (52.2)	75 (25.3)	67 (22.6)
*Lifetime sunburns*				
None	660	266 (40.3)	225 (34.1)	169 (25.6)
1 to 2	576	233 (40.5)	198 (34.4)	145 (25.2)
3 or more	1164	316 (27.2)	489 (42.0)	359 (30.8)

*Numbers may not sum to the overall total due to missing data. Two individuals were missing education information, one was missing smoking, three were missing skin sensitivity and twenty-six were missing information about lifetime sunburns. They were excluded from analyses.

† Sun sensitivity was defined as the reaction to 1 hour of sun exposure the first time in the summer.

### Main Effects

We first tested the independent associations of *RNASEL* (rs486907) and *MIR146A* (rs2910164) with risk of SCC or BCC (Table 2). More than half of all participants carried at least one copy of the *RNASEL* variant A-allele, while the *MIR146A* variant C-allele was less common and present in approximately one-third of participants. We did not observe an overall increase or decrease in the ORs for SCC or BCC among individuals carrying either of the *RNASEL* or *MIR146A* variants compared with those who were wild type at these loci. However, in an analysis of main effects stratified by sex, women who carried the *MIR146A* variant C-allele had a reduced odds ratio for BCC (OR_GC_ = 0.73, [95% CI = 0.52–1.03]; OR_CC_ = 0.29, [95% CI = 0.14–0.61], p for trend <0.001), but the association was not observed in men (Table 3). Additionally, we observed lower, but not statistically significant, odds ratios for SCC for men carrying the *RNASEL* variant A- allele (OR_AG_ = 0.88, [95% CI = 0.65–1.19]; OR_AA_ = 0.68, [95% CI = 0.43–1.08], p for trend = 0.10) compared to men who were wild type; but this was not observed in women (Table 3).

**Table 2:** Main effects of *RNASEL* rs486907 and *MIR146A* rs2910164 genotypes on non-melanoma skin cancer risk.

		BCC		SCC	
	Controls	N	OR (95% CI)*	N	OR (95% CI)*
***RNASEL***					
G/G	288	370	reference	258	reference
G/A	362	375	0.85 (0.68–1.06)	271	0.84 (0.66–1.07)
A/A	106	113	0.88 (0.64–1.21)	74	0.79 (0.56–1.13)
trend#			0.91 (0.79–1.06)		0.88 (0.74–1.04)
			*P for trend = 0.23*		*P for trend = 0.12*
***MIR146A***					
G/G	481	567	reference	388	reference
G/C	271	281	0.88 (0.71–1.10)	241	1.09 (0.87–1.38)
C/C	43	44	0.80 (0.51–1.26)	34	0.90 (0.55–1.48)
trend#			0.89 (0.75–1.05)		1.03 (0.85–1.23)
			*P for trend = 0.17*		*P for trend = 0.79*

*Adjusted for age, sex, level of education, cigarette smoking status 1 year before the reference date (for SCC only), skin sensitivity (measured by skin reaction after 1 hour of sun exposure the first time in the summer) and the number of lifetime painful sunburns.

# Trend for any variant allele.

**Table 3:** Main effects of *RNASEL* rs486907 and *MIR146A* rs2910164 genotypes on non-melanoma skin cancer risk stratified by sex.

	Controls		BCC				SCC			
	Men	Women	Men		Women		Men		Women	
	N	N	N	OR (95% CI)*	N	OR (95% CI)*	N	OR (95% CI)*	N	OR (95% CI)*
***RNASEL***										
G/G	182	106	215	reference	155	reference	166	reference	92	reference
										
G/A	212	150	214	0.90	161	0.79	175	0.88	96	0.77
				(0.68–1.20)		(0.56–1.12)		(0.65–1.19)		(0.51–1.16)
A/A	67	39	56	0.76	57	1.09	43	0.68	31	0.91
				(0.50–1.16)		(0.66–1.80)		(0.43–1.08)		(0.50–1.64)
trend#				0.88		0.98		0.84		0.90
				(0.72–1.07)		(0.77–1.23)		(0.68–1.04)		(0.68–1.19)
				*P-trend = 0.19*		*P-trend = 0.85*		*P-trend = 0.10*		*P-trend = 0.46*
***MIR146A***										
G/G	302	179	309	reference	258	reference	248	reference	140	reference
										
G/C	165	106	164	0.99	117	0.73	160	1.15	81	1.02
				(0.75–1.32)		(0.52–1.03)		(0.86–1.53)		(0.69–1.52)
C/C	19	24	31	1.62	13	**0.29**	21	1.13	13	0.76
				(0.88–2.98)		**(0.14–0.61)**		(0.57–2.23)		(0.35–1.63)
trend#				1.11		**0.64**		1.11		0.94
				(0.89–1.39)		**(0.49–0.83)**		(0.88–1.41)		(0.70–1.27)
				*P-trend = 0.34*		***P-trend <0.001***		*P-trend = 0.38*		*P-trend = 0.68*

*Adjusted for age, level of education, cigarette smoking status 1 year before the reference date (for SCC only), skin sensitivity (measured by skin reaction after 1 hour of sun exposure the first time in the summer), and the number of lifetime painful sunburns.

# Trend for any variant allele.

### Gene-gene Interaction Effects

We then assessed whether the presence of both variants influenced the risk of BCC or SCC (Table 4). Among those wild type for *MIR146A*, we did not observe a change in risk for individuals who carried a *RNASEL* variant A-allele (OR_BCC_ = 1.07, [95% CI = 0.82–1.41]; OR_SCC_ = 1.02, [95% CI = 0.75–1.39]), when compared to those that were wild type, whereas, among those who carried a *MIR146A* variant C-allele, those with a variant allele of in *RNASEL* had reduced odds of both BCC (OR = 0.57, [95% CI = 0.40–0.80], p for interaction = 0.005) and SCC (OR = 0.56, [95% CI = 0.38–0.81], p for interaction = 0.012), when compared to those who were wild type for *RNASEL*.

**Table 4:** Gene-gene interaction of *RNASEL* and *MIR146A* in relation to non-melanoma skin cancer risk.

Genotypes		Controls	BCC		SCC	
*MIR146A*	*RNASEL*	N	N	OR (95% CI)*	N	OR (95% CI)*
G/G	G/G	177	218	reference	137	reference
	G/A or A/A	259	310	1.07 (0.82–1.41)	204	1.02 (0.75–1.39)
G/C or C/C	G/G	96	140	reference	114	reference
	G/A or A/A	195	162	**0.57 (0.40–0.80)**	129	**0.56 (0.38–0.81)**
				***P-interaction = 0.005***		***P-interaction = 0.012***

*Adjusted for age, sex, level of education, cigarette smoking status 1 year before the reference date (for SCC only), skin sensitivity (measured by skin reaction after 1 hour of sun exposure the first time in the summer), and the number of lifetime painful sunburns.

## Discussion

In our population-based, case-control study of BCC and SCC, we found evidence of interacting effects of common variants in two genes involved in aspects of inflammation and immunity, *RNASEL* and *MIR146A*, on risk of NMSCs. While neither of these variants appeared to affect risk of BCC or SCC when considered singly in the entire population, gender-specific associations were observed, i.e. significant reduction in risk of BCC in women who carried the *MIR146A* variant C-allele, and a borderline reduction in risk of SCC in men who carried the *RNASEL* variant A-allele. This is consistent with our prior work indicating gender-specific immunogenetic risk effects for NMSCs, which reported that while skin type and lifetime number of sunburns were important risk factors for SCC and BCC in both men and women, the relative contribution of genetic variants involved in UV-induced immunosuppression to risk of SCC and BCC vary by sex [42], [43].

The rs486907 *RNASEL* variant has been associated with increased risk of several cancers [21], [25]–[32]. This Arg to Gln variant has been shown to inhibit dimerization of RNASEL into its active form, resulting in a 3-fold reduction in enzyme activity that strongly affects its endonuclease capacity and thus its pro-apoptotic activity [21]. However, there are inconsistencies across studies with regard to risk direction for rs486907. Studies of sporadic prostate cancers have shown that the variant A allele of rs486907 may be associated with lower grade tumors, as assessed by Gleason score [28], [44]. It is possible that the reduction in activity associated with rs486907 may have differential effects in various tissue contexts, including tumor types, and when found in combination with other genetic variants, such as *MIR146A* rs2910164.

RNASEL plays a significant role in viral clearance and it has been suggested that variation in *RNASEL* may alter risk of viral-associated cancers, such as head and neck squamous cell carcinoma and cervical cancer, as well as non-viral cancers such as breast cancer [45], [46]. Discrepancies between large studies of hereditary prostate cancer suggest that environmental factors, such as viral infection, may modulate the impact of *RNASEL* variation on carcinogenesis [18]. Indeed, viral infection with xenotropic murine leukemia virus-related virus (XMRV) has been observed to be more common in prostate cancers of individuals homozygous for the *RNASEL* rs486907 variant [47]. While *RNASEL* may be a more general marker of cancer risk, it is possible that *RNASEL* variants could also impact viral susceptibility, thus increasing the risk of developing a persistent infection with potentially oncogenic viruses such as human papillomavirus (HPV). As cutaneous HPVs have been previously associated with incidence of SCC, a future area of inquiry would be to examine this relationship according to *RNASEL* genotypes in our study population [48]–[51].

The immune system imposes highly regulated multi-level controls upon its responses to pathogen and miR-146a plays a key role in modulating these functions. While the inflammatory response is essential for clearing pathogenic infection, it must be tightly regulated–a role that is fulfilled in part by miR-146a in response to TLR4 activation [52]. As demonstrated in *MIR146A* knockout mice, miR-146a impacts both innate and adaptive immunity, with loss of miR-146a leading to hyper-responsiveness to LPS challenge, an activated T-cell phenotype, an overabundance of pro-inflammatory cytokines, and eventually to hematopoietic malignancy [53]. Interestingly, the heightened immune response characteristic of miR-146a knockout mice makes them more resistant to bacterial infection than wild-type animals [54]. *MIR146A* transcription is induced by the pro-inflammatory immune response and NF-kB activation and in turn, miR-146a targets NF-kB signaling component IRAK-1 and TRAF, creating a negative feedback loop to downregulate the immune response and preventing inflammatory damage [55]. In other words, inefficient binding of miR-146a to its targets in the NF-kB signaling pathway, such as IRAK-1, or diminished endogenous levels of miR-146a can both promote immune overactivation and inflammation [11], [56], [57]. Such effects have been shown to occur in the presence of rs2910164, which leads to diminished levels of mature miR-146a, which in turn relieves the inhibition of its targets in the cell [12], [13]. Therefore, the *MIR146A* rs2910164 variant would be hypothesized to impact the immune system by increasing immune hyper-responsiveness.

As a key regulator of inflammation, it is not surprising that *MIR146A* variation has also been implicated in oncogenesis and vascular endothelial activation, as well as other inflammatory and autoimmune diseases, including rheumatoid arthritis, psoriatic arthritis and systemic lupus erythematosus (SLE) [52], [56]–[59]. Specifically, rs2910164 has been associated with increased incidence of thyroid tumors [12], [13], [16]. A recent study in a Hungarian population found an association between rs2910164 and increased susceptibility to head and neck squamous cell carcinoma [60]. Others have reported similar associations between rs2910164 and cancers of the prostate, cervix, breast and digestive tract [15], [61]–[64]. However, a small body of work has begun to show that rs2910164 may be protective in some populations and in specific cancer types. One case-control study found that the rs2910164 variant actually reduced risk of colorectal cancer in a Chinese population [65]. A meta-analysis of 29 studies showed significantly lower cancer risks among Asians associated with rs2910164, while a tumor-type subgroup analysis showed that the rs2910164 C allele associated with decreased risk of hepatocellular carcinoma and cervical squamous cell carcinoma [66]. Another meta-analysis reported that rs2910164 was associated with reduced cancer risk in Caucasians, but with increased risks in certain subgroups such as Asians, men, and smokers [67]. Similarly, it is possible that the underlying molecular phenotype and etiology of SCC and BCC may impact the activity of these immune regulatory SNPs, potentially accounting for risk reduction that we observed for these tumor types in our study. Further work is necessary to elucidate the specific factors that may additionally influence disease risk in the context of rs2910164 genetic variation.

In this study, a decrease in risk of skin cancer was observed only when both variants, rs486907 *RNASEL* and *MIR146A* rs2910164, were present. RNASEL is a predicted target of miR-146a [17], but currently, to our knowledge, direct evidence for miR-146a as a transcriptional regulator of *RNASEL* mRNA is lacking. Future work is needed to further clarify the regulatory relationship between miR-146a and *RNASEL.* If RNASEL is indeed targeted by miR-146a, the reduced levels of miR-146a due to the rs2910164 variant allele would likely result in the production of more of RNASEL, albeit in a less active form in the presence of rs486907, as this variant produces a truncated form of RNASEL with reduced functionality. In this scenario, one might predict a decreased or unchanged risk of cancer. However, given our results showing that the two variants together reduce NMSC risk, it is more plausible that these two immune modulators might be acting upon one another in a less direct manner and that the observed reduction in skin cancer risk in our study may result from independent changes that are predicted from each of these variants (i.e. altered immune function or impaired apoptosis). In this alternate scenario of an indirect relationship, the interaction between miR-146a and *RNASEL* may not only be dependent upon their expression levels, but also on the expression levels of other miR-146a target transcripts. A reduction in RNASEL activity in the presence of rs486907 would be predicted to increase susceptibility to viruses and impair apoptosis, and ultimately increase cellular stress and targeting by the immune system. Concomitantly, the *MIR146A* rs2910164 variant likely alters overall immune function and increases immune hyper-responsiveness. Little is known about how these two immune mediators may impact one another in the context of NMSC, but it is possible that the interaction between RNASEL and miR-146a might reflect the interaction between important cell types in the cutaneous epithelium, such as between T-cells, where miR-146a is critical to suppressor function, and keratinocytes, where RNASEL may play a role in viral defense or regulating the cellular stress response. An individual’s immune response or surveillance may be heightened in the presence of *MIR146A* rs2910164 and can better target cells that are stressed due to altered RNA levels or that are infected with a cutaneous virus, due to reduced RNASEL activity.

We observed some sex-specific differences in susceptibility, including a significant reduction in risk of BCC in women who carried the *MIR146A* variant C-allele, and a borderline reduction in risk of SCC in men who carried the *RNASEL* variant A-allele. We were unable to examine the SNP-SNP interaction for men and women separately, due to sample size limitations. In previous work, we observed sex differences in susceptibility to SCC and BCC, in relation to other immune-related genetic variants [42], [43]. Specifically, while common risk factors for SCC and BCC, such as skin type and lifetime number of sunburns, were important in both men and women, we found sex-specific differences in the relative contribution of genetic variants involved in UV-induced immunosuppression to risk of SCC and BCC [42], [43]. It is possible that estrogen could somehow play a role in these observed differences in NMSC risk. Estrogen receptors, specifically estrogen receptor-β, are expressed in human keratinocytes, and estrogen can impact proliferation of keratinocytes, wound healing and vascularization of skin [68]. Interestingly, interferon-stimulated exonuclease gene 20 kDa (ISG20), which is member of the 3′ to 5′ exonuclease family that also includes RNASEL, can be induced by both interferon and estrogen [69], [70]. Although very speculative, one could envision a mechanism in which it is possible that RNASEL could also be dually-regulated by interferon and estrogen signaling, providing a link between gender and the RNASEL SNP.

The strength of our study lies in its population-based, case-control design, the large number of histologically confirmed cases of SCC and BCC identified through a surveillance network of dermatologists, dermopathologists and pathologists, as well as the availability of covariate data on lifestyle factors and skin characteristics, such as sun exposure and skin sensitivity. While this population-based design is representative of the general population and less susceptible to selection bias than hospital and clinic-based studies, we cannot rule out the possibility that non-participation introduced selection bias or residual confounding might exist. Further, our study has the potential for lack of generalizability due to the fact that it is located at higher latitude relative to other at-risk populations.

To our knowledge, our study is the first to examine the effects of *RNASEL* and *MIR146A* genetic variants on non-melanoma skin cancer susceptibility. Our findings suggest that polymorphisms in these immune and inflammatory regulators may influence susceptibility to non-melanoma skin cancers. Further, our work is among the first to suggest a SNP-SNP interaction for a miRNA and its target gene. These data imply that RNASEL, an enzyme involved in cellular and viral RNA turnover, is controlled by miR-146a and that this process may be important in skin cancer etiology.
